# An Optimized and Efficient CRISPR/Cas9 System for the Endophytic Fungus *Pestalotiopsis fici*

**DOI:** 10.3390/jof7100809

**Published:** 2021-09-28

**Authors:** Xinran Xu, Runye Huang, Wen-Bing Yin

**Affiliations:** 1 State Key Laboratory of Mycology, Institute of Microbiology, Chinese Academy of Sciences, Beijing 100101, China; xuxr@im.ac.cn (X.X.); huangrunye20@mails.ucas.ac.cn (R.H.); 2 Savaid Medical School, University of Chinese Academy of Sciences, Beijing 100049, China

**Keywords:** endophytic fungi, CRISPR/Cas9, genetic manipulation, *Pestalotiopsis fici*, dual-locus genome editing

## Abstract

Endophytic fungi are emerging as attractive producers of natural products with diverse bioactivities and novel structures. However, difficulties in the genetic manipulation of endophytic fungi limit the search of novel secondary metabolites. In this study, we improved the polyethylene glycol (PEG)-mediated protoplast transformation method by introducing the CRISPR/Cas9 system into endophytic fungus *Pestalotiopsis fici*. Using this approach, we performed genome editing such as site-specific gene insertion, dual-locus mutations, and long DNA fragment deletions in *P. fici* efficiently. The average efficiency for site-specific gene insertion and two-site gene editing was up to 48.0% and 44.4%, respectively. In addition, the genetic manipulation time with long DNA fragment (5–10 kb) deletion was greatly shortened to one week in comparison with traditional methods such as *Agrobacterium tumefaciens*-mediated transformation (ATMT). Taken together, the development of the CRISPR/Cas9 system in the endophytic fungus will accelerate the discovery of novel natural products and further biological study.

## 1. Introduction

Endophytic fungi reside in the internal tissues of living organisms at some stages in their life cycle without causing any symptoms, and have currently been acknowledged as one of the most promising sources of bioactive compounds. Those bioactive metabolites have diverse structural features to adapt to the unique living environment, some of which have metabolites that display significant pharmaceutical and ecological properties [1]. The genus *Pestalotoiopsis* belongs to the *Amphisphaeriaceae* (*Coelomycetes*) family, which has been extensively isolated from healthy plant tissues and has been considered a main part of endophytic fungi in the past decade [2]. Many fungi in this genus can produce diverse natural products with good biological activity, for instance, *P. versicolor* and *P. microspora*, which produce Taxol more efficiently than the one found in the earlier reported fungus *Taxomyces andreanae* [3,4]. Moreover, a wide variety of compound types has been reported from *Pestalotiopsis* spp., making this genus a particularly rich source for bioprospecting, thus inviting much attention from the scientific community [5,6].

*Pestalotiopsis fici* was reported as a genius producer of natural products. For example, 88 natural products were identified from this species, including 70 new compounds [7]. Among them, chloropupukeananin was the first derivative of chlorinated pupukeanane found in fungi [8]. Compounds such as chloropupukeananin, chloropestolide A, chloropupukeanolide A and siccayne showed cytotoxic activity against human cancer cell lines [9,10,11,12,13,14,15], and pestalofones C and E exhibited good anti-fungal activity on *Aspergillus fumigatus* [12]. Due to the richness in metabolites, *P. fici* was sequenced [16] and 76 biosynthetic gene clusters (BGCs) were found using bioinformatic analysis [17]. These gene clusters include 30 polyketide synthases (PKSs), 14 nonribosomal peptide synthases (NRPSs), 16 NRPS-like enzymes, 12 terpene synthases (TSs) as well as 4 hybrid NRPS-PKSs. Except for the BGCs of pestheic acid [18], iso-A82775C [19] and 1,8-dihydroxynaphthalene (DHN) melanin [20], the function of the other gene clusters remains unknown. The main barrier is low efficiency of genetic manipulation in *P. fici*.

Previously, an *Agrobacterium tumefaciens-*mediated transformation (ATMT) method was established in *P. fici*. The biosynthetic gene clusters of pestheic acid and iso-A82775C were identified by using the ATMT method [18,19]. The ATMT method can be applied in fungal cells independently of their growth stages but involves multiple complicated manipulation steps and requires 14−17 days to complete one round of transformation. Therefore, in order to shorten the experimental time and improve efficiency, a PEG-mediated protoplast transformation method in *P. fici* was established, which improved protocol, and only took 7−10 days to complete one round of transformation [17]. In previous studies, many epigenetic factors [17,21] and regulatory genes [22] have been successfully knocked out using this method. However, the transformation rates were not high, and it is necessary to screen a large number of transformants. In some cases, it is very difficult to obtain gene mutations when genes locate in special positions of the chromosome [23]. Therefore, we optimized the method and improved the efficiency of the genetic manipulation system.

The clustered regularly interspaced short palindromic repeats (CRISPR)/CRISPR-associated protein (Cas) system has been established in some filamentous fungi, such as *Trichoderma reesei* [24], *Penicillium chrysogenum* [25], *A. fumigatus* [26], *A. niger* [27] and *Glarea lozoyensis* [28]. Most commonly used is the CRISPR/Cas9 system from *Streptococcus pyogenes*, which consists of Cas9 protein and two RNAs—CRISPR RNA (crRNA) and trans-activating crRNA (tracrRNA) [29,30]. First, Cas9 assembles the mature single guide RNA (sgRNA), which is composed of crRNA- and tracrRNA-derived sequences connected by an artificial tetraloop, into an effector complex. The complex is capable of recognizing target DNA guided by a 20-nucleotide (nt) guide sequence on 5’ sgRNA. The Cas9-sgRNA complex specifically cleaves double-stranded DNA (dsDNA) and generates a double-stranded break (DSB) in the target region, which greatly increases the rate of homologous recombination. Based on the characteristics of Cas9, we established a CRISPR/Cas9-based efficient genetic manipulation system in the endophytic fungus *P. fici*. As proof of principle, we performed genome site-specific gene insertion, gene deletion and dual-locus mutations in *P. fici*. The results showed that the CRISPR/Cas9 system in *P. fici* not only improved the efficiency of gene manipulation, but also greatly reduced the time required for one round of transformation.

## 2. Materials and Methods

### 2.1. Strain, Media and Culture Conditions

*Pestalotiopsis fici* CGMCC3.15140 and its transformants were grown on Potato Dextrose Agar (PDA) or Potato Dextrose Broth (PDB) with appropriate antibiotics as required at 25 °C. *Escherichia coli* strain DH5a was cultured in an LB medium with antibiotics appropriate for the resistance markers on the plasmid DNA. The Cas9 open reading frame (ORF) was amplified from the commercial vector pX330, which was ligated to protein expression vector pET28a with two His-tags.

### 2.2. Synthesis and Purification of Single Guide Sequence (sgRNA)

To generate the sgRNA, we used an in vitro transcription based on the T7 RNA polymerase strategy as described previously [31]. Briefly, the CRSIPR design tool [32] was used to identify CRISPR single guide sequences within the two 250 bp nearest to the hooks. Primers used for the synthesis of sgRNA are listed in Supplementary Table S1. A double-stranded transcription template was prepared by amplifying a single-stranded oligonucleotide; the three primers required for PCR amplification were antisense template oligonucleotide with the T7 promoter sequence, sgRNA-F and sgRNA-R. The DNA templates were purified by DNA Clean and Concentrator Kits (Zymo Research, Irvine, CA, USA). In vitro transcription was performed with the T7 High Efficiency Transcription Kit (Transgen, Beijing, China). After transcription, large quantities of RNA were purified by the EasyPure RNA Purification Kit (Transgen). Finally, the RNA was resolved in 30 μL of RNase-free water, and 1 μL was used to measure the concentration and purity of sgRNA by NanoDrop. A concentration of 300–1000 ng/μL and A260/280 > 2.0 was supposed to be ideal. In vitro, sgRNA would be prepared when ready to use.

### 2.3. Gene Cloning, DNA Cassettes Construction and Genetic Manipulation

The primers used are listed in Supplementary Table S1. PCR amplification was carried out with TransStart ^®^ FastPfu DNA polymerase (Transgene Biotech, Telangana, India) on a T100™ Thermal cycler (Bio-Rad, Hercules, CA, USA). The screening of PCRs was performed by a 2× Taq Mix kit (TIANGEN BIOTECH, Beijing, China) after transformation. The amplified fragment for fungal transformation was purified with DNA Clean and Concentrator Kits (Zymo Research, USA).

For the construction of Cas9 vectors, the amplified *gpdA::Cas9* ORF was inserted into SpeI-AscI sites of binary vector pAg1-H3 to give pAg1-gpdA-Cas9 [23]. To generate the insertion and deletion cassettes, we used double-joint PCR procedures [33]. For the construction of deletion cassettes, the 1 kb fragment upstream and downstream of target genes was amplified from the genomic DNA of *P. fici* using the designated primers. The marker gene *neo* (2059 bp) was amplified with the plasmid pAg1-H3-G418. The three amplified PCR products were purified with a Zymoclean Gel DNA Recovery Kit, quantified, and fused with double-joint PCR procedures. For the construction of insertion cassettes, *gpdA* sequence was amplified from plasmid pWY25.16 [34], the 1 kb fragment upstream and downstream of target sites was amplified from the genomic DNA of *P. fici*, and the marker gene *neo* (2059 bp) was amplified with the plasmid pAg1-H3-G418. These four amplified PCR products were fused by double-joint PCR.

### 2.4. Transformations of P. fici

We made some changes to PEG-mediated transformation methods of *P. fici* which were described previously [17]. Briefly, the mycelium of *P. fici* was harvested and ground by TissueLyser with 500 µL of PDB media and two steel beads, and then transferred into 30 mL of TG medium (10% glucose (*w*/*v*), 1% tryptone (*w*/*v*)) and further incubated at 25 °C for up to 12 h (200 r.p.m. min^−1^). The mycelium was harvested by filtration and washed with N-M solution (0.3 M NaCl, 0.3 M MgSO_4_, pH was adjusted to 7.5–7.8 with 1 M Tris-HCl (pH 7.5)). The harvested mycelia were transferred to a lysing solution (lysing enzymes (30 mg mL^−1^), dissolved in 10 mL of N-M solution) and the mixture was gently shaken at 25 °C for 6 h (100 r.p.m. min^−1^). Protoplasts were purified by filtration through a sterile miracloth (Merck), collected at 5000 r.p.m for 15 min at 4 °C and washed twice with N-M solution. Finally, the protoplasts were suspended in 0.6 M KCl at a concentration of 10^7^–10^8^ mL^−1^.

Approximately 1.0 × 10^7^ protoplasts in 100 μL of 0.6 M KCl were mixed with 5 μg of purified DNA fragment, in vitro synthetic sgRNA and 50 μL of 25% PEG (25% PEG3350/4000, M/V, 0.6 M KCl, 50 mM CaCl_2_, pH was adjusted to 7.5–7.8 with 1 M Tris-HCl, pH 7.5) in a 1.5 mL centrifuge tube and chilled on ice for 30 min. Then, 1 mL of 25% PEG was added to the mixture and swirled gently, followed by incubation at room temperature for 30 min. The mixture was added to 100 mL of PDSSA medium (0.6 M sucrose, 1% honey (*v*/*v*), 3.9% PDA (*w*/*v*)), mixed well and split into 6 plates, followed by incubation at 25 °C =overnight. After its germination, a PDA medium containing antibiotics (hygromycin B and G418) was used to overlay the bottom plate, and grown at 25 °C for 2 days.

Transformants were transferred to PDA plates containing hygromycin B and G418 and subcultured twice to ensure hereditary stability. The mycelia were transferred to a 60 mm plate containing 4 mL of PDB liquid medium for genomic DNA extraction. The selected mutants were tested by diagnostic PCR using primers listed in Supplementary Table S1.

## 3. Results

### 3.1. Development of a Cas9-Dependent Genome Editing System for P. fici

In order to apply the CRISPR/Cas9 system in *P. fici*, we constructed a plasmid named pAg1-gpdA-Cas9, which contained the hygromycin B resistance gene and *cas9* coding sequence from a commercially available plasmid pX330. The 5’ and 3’ ends of the *cas9* gene were linked with a nuclear localization sequence (NLS), which helped the Cas9 protein to be located in the nucleus. This *cas9* gene sequence had been shown to be suitable for filamentous fungi [27]. Then, we transformed the plasmid pAg1-gpdA-Cas9 into WT protoplast to obtain the original strain of CRISPR/Cas9 system TYPZ36.

**Figure 1:**
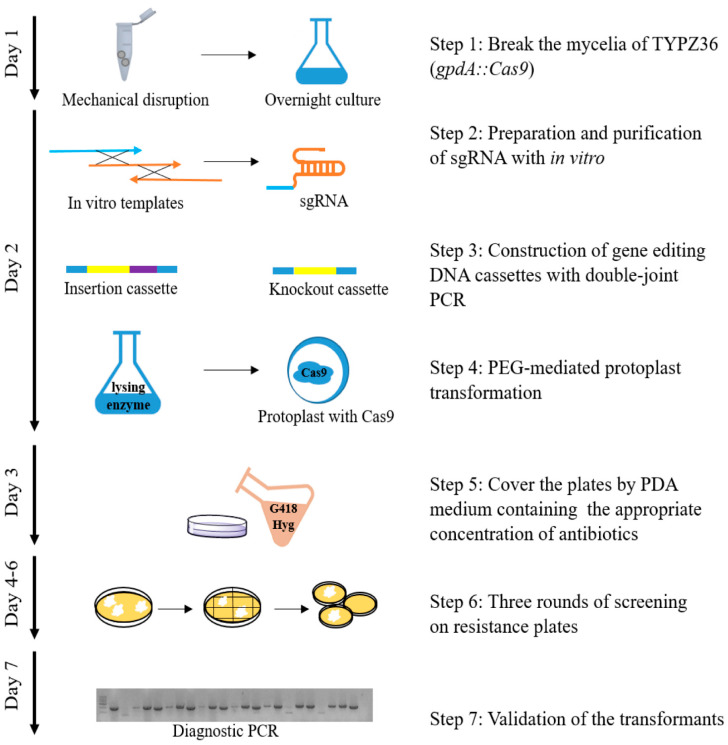
Construction of CRISPR/Cas9-based genetic manipulation system. This method is based on PEG-mediated protoplast transformation method and CRISPR/Cas9 system. The mycelium of *P. fici* was broken and cultured overnight to obtain fresh and tender mycelia. sgRNAs were synthesized in vitro by T7 RNA polymerase strategy and purified by EasyPure RNA Purification Kit. The insertion or deletion cassettes were constructed by double-joint PCR. The tender mycelia were digested by lysing enzyme and made into protoplasts. After that, targeted DNA cassettes and sgRNAs were co-transformed into protoplasts. The plates were covered by PDA medium containing the appropriate concentration of antibiotics on the next day, followed by three rounds of screening to obtain the transformants. Finally, transformants were screened by diagnostic PCR. The entire process of CRISPR/Cas9-based genetic manipulation takes 7 days.

Before protoplast transformation, a three-part preparation process needed to be completed. Firstly: sgRNAs were designed based on a CRSIPR design tool [32] and synthesized in vitro by T7 RNA polymerase strategy. Two sgRNAs were needed for gene knockout and only one sgRNA was sufficient for overexpression. Secondly, insertion and deletion cassettes were constructed with double-joint PCR. Thirdly, the protoplasts were prepared by the digestion of tender mycelia with lysing enzyme. During protoplast transformation, sgRNA and DNA cassettes were mediated into the protoplast via PEG, and sgRNA guided Cas9 to cleaved double-stranded DNA in target sites. At the same time, the insertion or deletion cassettes with homologous arms achieved an efficient homologous recombination in case the DNA double-stranded broke down. After protoplast transformation, three rounds of screening were performed to get as many homozygous as possible. The screening marker on both the gene deletion cassettes and insertion cassettes was G418, and both hygromycin B and G418 resistant colonies were selected and identified by diagnostic PCR (Figure 1).

### 3.2. DNA Fragment Insertion in CRISPR/Cas9-Dependent Genome Editing System in P. fici

Gene fragment insertion is a conventional molecular biological method. Site-specific gene fragment insertion can mediate the transcriptional activation and inhibition of specific genes, and the expression of foreign genes and gene clusters. For the un-engineered endophytic fungus *P. fici*, the efficiency of site-specific gene editing by homologous recombination was very low. In this study, Cas9-mediated DNA double-stranded breaks activated the homology-directed repair (HDR) mechanism in *P. fici* in the presence of homologous arms of the target site, enabling efficient site-specific homologous recombination. For the target insertion sites, a total of four specific genomic loci from synthetic gene clusters of *P. fici* were selected. We inserted the *gpdA* sequence (1.4 kb) and the screening marker *neo* sequence (2.1 kb) in front of the target sites when verifying the insertion efficiency of the CRISPR/Cas9 genome editing system in *P. fici*.

**Figure 2:**
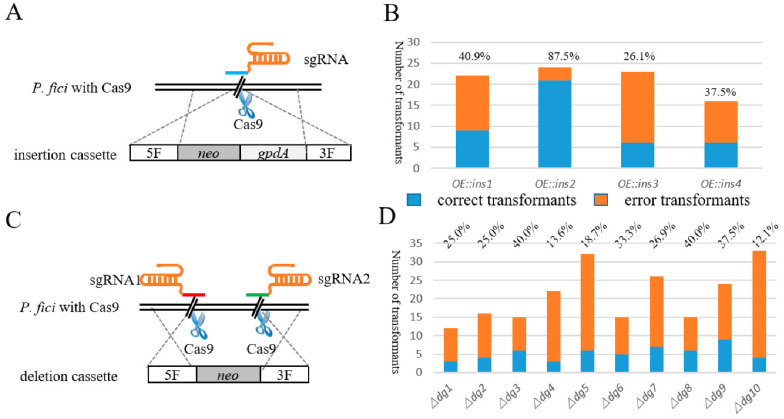
Schematic illustration and efficiency of single-locus genome editing. (**A**) Schematic of gene insertion mediated by Cas9, sgRNA and insertion cassette. (**B**) The insertion efficiency of CRISPR/Cas9-based genetic manipulation system. The blue column represents the number of correct transformants, and orange column represents the number of incorrect transformants. (**C**) Schematic of gene knockout mediated by Cas9, sgRNA and deletion cassette. (**D**) The knockout efficiency of CRISPR/Cas9-based genetic manipulation system. The blue column represents the number of correct transformants, and orange column represents the number of incorrect transformants.

The insertion cassettes were constructed with double-joint PCR, including 1 kb upstream and downstream of the insertion site, screening marker *neo* and *gpdA* sequence (Figure 2A). The total amount of insertion cassettes used for transformation was 5 μg. Two principals were applied in designing sgRNA: one was that the PAM site was close to the ATG and the other was that RNA had a high website score. The in vitro synthesized sgRNA was transformed into the protoplast of *P. fici* together with the insertion cassette. Hygromycin B and G418 acted as two screening markers to screen the correct transformants. For gene insertion, 22, 24, 23, 16 transformants of 4 insertion loci were selected from the 2 resistance plates; after PCR screening, the number of correct transformants were 9, 23, 6 and 6, respectively, and the average correctness rate was 48.0% (Figure 2B). Although the insertion took place in four sites with the same DNA fragment, one of these sites, namely *ins2*, had a higher insertion efficiency compared to the other three sites, which may be related to the location of the target locus on the chromosome. Excluding the efficiency of the *ins2* site, the average insertion efficiency of the other three sites was 34.8%, which was also much higher than direct gene manipulation in the WT.

### 3.3. DNA Deletion in CRISPR/Cas9-Dependent Genome Editing System in P. fici

Gene deletion is an essential molecular method to study gene function. Due to the complex chromosomal composition of filamentous fungi, it is difficult to perform large fragment gene deletion in filamentous fungi. Currently, most applications of the Cas9 system in filamentous fungi focus on inactivation of genes by activating the non-homologous end-joining (NHEJ) mechanism, resulting in possible base insertion or deletion, or knocking out the target gene with a length of 1–2 kb. There are still difficulties for gene deletion with a length of 5–10 kb, which commonly appear in the secondary metabolite synthesis gene clusters.

We knocked out the core genes from 10 biosynthetic gene clusters, and their sizes were distributed from 5 to 10 kb. The deletion cassettes were also constructed by the double-joint PCR (Figure 2C), the connected segments were 1 kb upstream and downstream of the target knockout sequence and the screening marker was gene *neo*, the total amount of the knockout cassette was 5 μg. Unlike gene insertion, we designed two Cas9 cutting sites upstream and downstream of the target gene to knock out the long DNA fragments, because two in vitro sgRNAs and one deletion cassette were needed during PEG-mediated protoplast transformation. As illustrated in Table 1 and Figure 2D, this system functioned well; the maximum deletion efficiency was up to 40.0% and the average efficiency was 27.2%. Our results showed that the Cas9 system could universally knock out the 5–10 kb gene fragments, in which the deletion efficiency had no significant correlation with the fragment length.

### 3.4. Dual-Locus Editing in CRISPR/Cas9-Dependent Genome Editing System in P. fici

**Figure 3:**
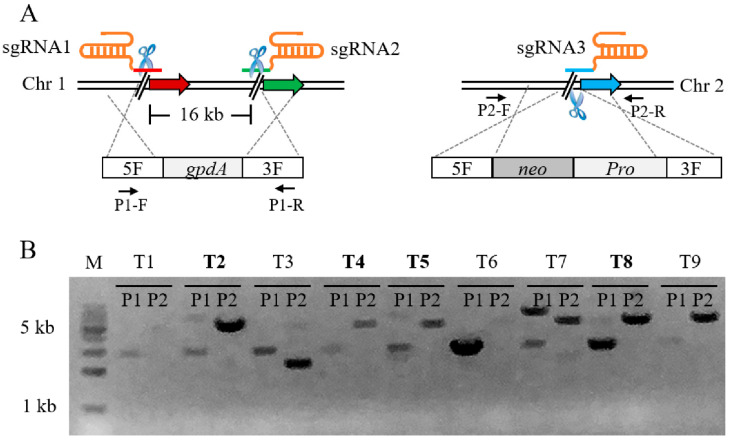
Schematic illustration and verification of dual-locus genome editing. (**A**) Schematic of dual-locus genome editing mediated by Cas9, 3 sgRNAs and 2 donor DNA cassettes. (**B**) PCR analysis of selected transformants. The pairs of primers P1-F/R and P2-F/R were used to amplify the mutants; the expected fragments were 2569 bp and 4990 bp. The mutants highlighted in bold were correct.

Since the Cas9-dependent genome editing system was highly effective for single-locus editing, we attempted to edit two loci at different chromosomal locations with one screening marker. As a proof of principle, we constructed an insertion cassette to knock out the gene *gd11* on a chromosome while inserting *ins5* which was adjacent to *gd11*, and another insertion cassette was simultaneously inserted at the target site *ins6* on another chromosome (Figure 3A). In this process, three sgRNA are required to co-operate with Cas9, with only one resistance screening tag required. A total of three sgRNA and two DNA cassettes were co-transformed into protoplasts of pYPZ36. As expected, after screening with selective plates containing G418 and hygromycin B, we obtained correct transformants, and the efficiency of simultaneous dual-locus genome editing was up to 44.4% (Figure 3B). This result demonstrated that the Cas9-dependent genome editing system can not only perform single locus gene editing, but can also perform a further edit of two or even multiplex locus simultaneously. On the one hand, dual loci were edited simultaneously in one round of the experiment, which greatly saved time; on the other hand, additional resistance screening markers were not introduced, reducing the unnecessary economic losses. In this experiment, the knockout DNA fragment was up to 16 kb, implying that the Cas9-dependent genome editing system had the potential to knock out large gene fragments efficiently.

## 4. Discussion

Establishing efficient genetic operating systems in endophytic fungi is crucial to further in-depth research. The CRISPR/Cas9 system has been developed into a set of powerful tools for manipulating the filamentous fungi genome [24,25,26,27,28]. In this study, a CRISPR/Cas9-dependent genome editing system was successfully developed in the endophytic fungus *P. fici*. To our knowledge, this is the first report on a successful induction of the CRISPR/Cas9-based genetic modifications in endophytic fungi. Using this system, we successfully achieved single-locus genome editing, including site-specific gene insertion and long DNA fragment (5–10 kb) deletions, with accuracy rates as high as 27.2% and 48.0%, and we also accomplished simultaneous dual-locus genome editing of different loci with only one selection marker integration via a single transformation. The experimental time required for one round of transformation was only half the time of the traditional ATMT method.

It was previously reported that in the PEG-mediated protoplast transformation method, the Ku70–Ku80 heterodimer and the DNA ligase IV (Lig4)–Xrcc4 complex [35] were often knocked out to improve homologous recombination. This protocol required multiple genetic manipulations, which did not provide benefits for endophytes, because endophytes may undergo degeneration after multiple genetic manipulations. The CRISPR/Cas9-dependent genome editing system not only improves the efficiency of homologous recombination, but also can edit multiple loci in a single transformation, which greatly reduces the number of genetic manipulations. In addition, the mutants edited by the CRISPR/Cas9-dependent genome editing system were grown faster than a wild-type strain. Although the phenomenon was unaccounted for, this was beneficial and harmless for slow-growing endophytic fungi. Furthermore, the CRISPR system is varied and suitable for upgrades to the genetic operating system [36,37]. The fusion of various protein motifs to Cas effector proteins has facilitated a diverse set of genetic manipulations, such as base editing, transposition, recombination, and epigenetic regulation [38,39,40,41,42,43].

For a novel species, an efficient genetic operating system is more conducive to the study of its growth and development models [22,44]. It also facilitates the underlying gene cluster activation and identification in original strains, such as using genetic operation to knock out or overexpress epigenetic regulatory factors [17,21], global regulators [45], pathway-specific transcription factors [46,47,48,49], core genes or even the whole-gene clusters [50,51,52,53,54,55,56], leading to the production or disappearance of compounds. Meanwhile, chassis construction has always been the core for synthetic biology and green biomanufacturing of natural product. *P. fici* is one of the few plant endophytes for which a genetic operation system has been established. These advantages can help it become a heterologous expression host for the production of valuable compounds.

In conclusion, the construction of an efficient CRISPR/Cas9-dependent genome editing system in *P. fici* can not only be used to further study this fungus, but also used as a model strain of similar fungal genus to excavate more secondary metabolites. While assisting in unlocking the CRISPR toolset in *P. fici*, CRISPR/Cas9-mediated gene manipulation methods of *P. fici* are capable of fast and efficient genomic manipulation. This approach can provide a general method for the establishment of genetic systems in fungi.

## Figures and Tables

**Table 1:** Description of the genes that were knocked out.

Name	Length (bp)	Amino Acid (aa)	Transformants	Verified Transformants	Efficiency
gd1	5458	1796	12	3	25.0%
gd2	5802	1933	16	4	25.0%
gd3	7110	2209	15	6	40.0%
gd4	7134	2313	22	3	13.6%
gd5	7250	2269	32	6	18.7%
gd6	8031	2568	15	5	33.3%
gd7	8085	2536	26	7	26.9%
gd8	8139	2600	15	6	40.0%
gd9	8512	2583	24	9	37.5%
gd10	9133	2969	33	4	12.1%

## Data Availability

The data presented in this study are available in this manuscript and can be requested from the corresponding author.

## Supplementary Materials

The following are available online at https://www.mdpi.com/article/10.3390/jof7100809/s1, Figure S1: PCR identification of gene insertion mutants, Figure S2: PCR identification of gene knockout mutants, Table S1: primers used in this study.
