# Supplementary material for: An Optimized and Efficient CRISPR/Cas9 System for the Endophytic Fungus Pestalotiopsis fici
Source: J Fungi (Basel). 2021 Sep 28;7(10):809. doi: 10.3390/jof7100809 (PMC8539907; doi:10.3390/jof7100809)
Supplement: Supplementary file 1 [file jof-07-00809-s001.zip › jof-1393770-supplementary.pdf]

Supplementary Information for

# An Optimized and Efficient CRISPR/Cas9 System for the Endophytic Fungus *Pestalotiopsis fici*

Xinran Xu[1,2], Runye Huang[1,2] and Wen-Bing Yin [1, 2,*]

1 State Key Laboratory of Mycology, Institute of Microbiology, Chinese Academy of Sciences, Beijing 100101, China; 15600660637@163.com (X.X.); huangrunye20@mails.ucas.ac.cn (R.H.)

2 Savaid Medical School, University of Chinese Academy of Sciences, Beijing 100049, China

* Correspondence: yinwb@im.ac.cn; Tel.: +86-10-64806170

**Keywords:** Endophytic fungi; CRISPR/Cas9; genetic manipulation; *Pestalotiopsis fici*; dual-locus genome editing.

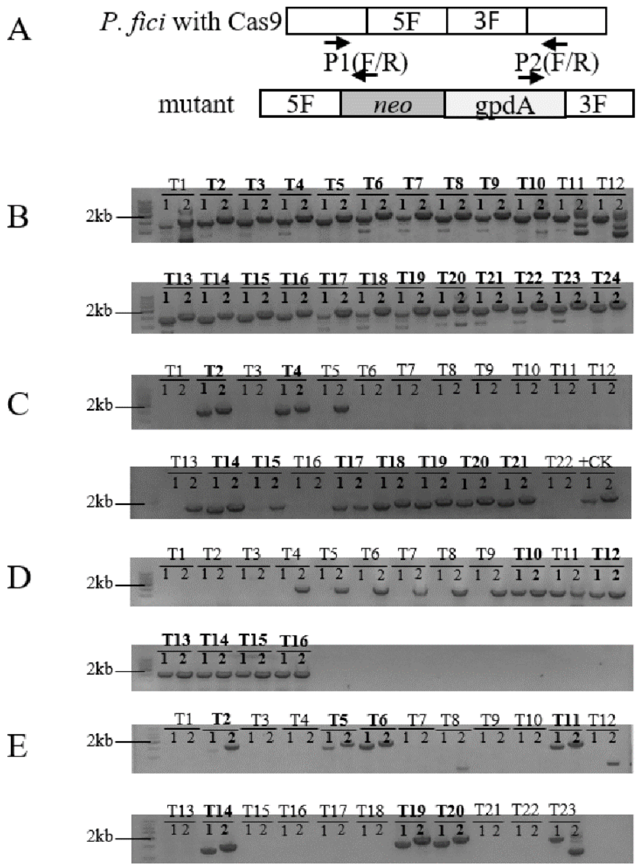


Figure S1 PCR identification of gene insertion mutants. The mutants highlighted in bold were correct.

A. Schematic of insertion cassette. The pairs of P1_F/R and P2_F/R were used to amplify the mutants. B. PCR identification of overexpression *ins2* mutants. C. PCR identification of overexpression *ins1* mutants. D. PCR identification of overexpression *ins4* mutants. E. PCR identification of overexpression *ins3* mutants.

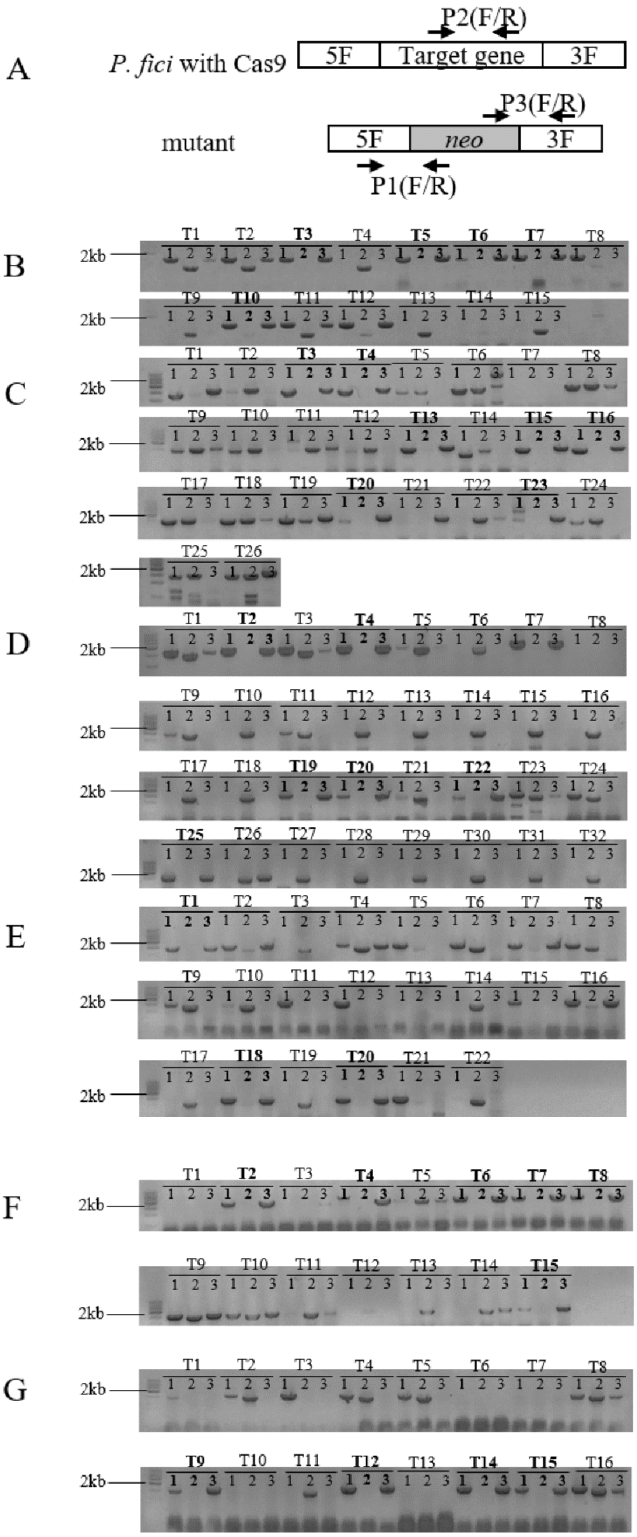
A
P. fici with Cas9
5F
Target gene
3F
P2(F/R)
mutant
5F
neo
3F
P3(F/R)
P1(F/R)
B
C
D
E
F
G
2kb

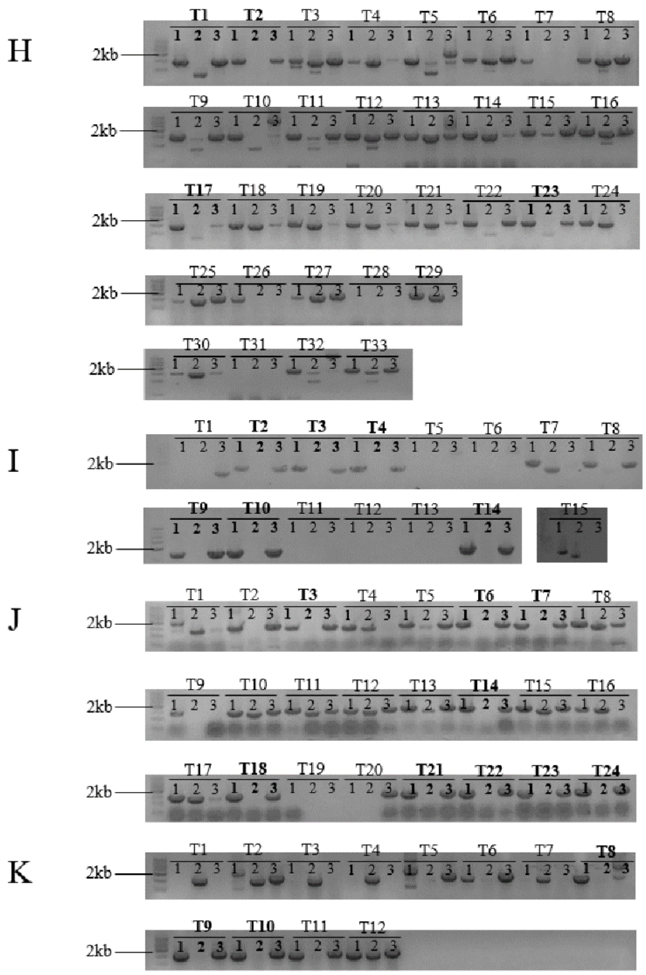


Figure S2 PCR identification of gene knockout mutants. The mutants highlighted in bold were correct.

A. Schematic of deletion cassette. The pairs of primers P1_F/R and P3_F/R were used to amplify the mutants, the pair of P2_F/R was used to amplify the target knockout gene. B. PCR was carried out to confirm the deletion of the *gd6*. C. PCR was carried out to confirm the deletion of the *gd7*. D. PCR was carried out to confirm the deletion of the *gd5*. E. PCR was carried out to confirm the deletion of the *gd4*. F. PCR was carried out to confirm the deletion of the *gd3*. G. PCR was carried out to confirm the deletion of the *gd2*. H. PCR was carried out to confirm the deletion of the *gd10*. I. PCR was carried out to confirm the deletion of the *gd8*. J. PCR was carried out to confirm the deletion of the *gd9*. K. PCR was carried out to confirm the deletion of the *gd1*.

Table S1 Primers used in this study

| Primers | Oligonucleotide sequence (5'-3') | Uses |
| --- | --- | --- |
| sgRNA-F | GTTTTAGAGCTAGAAATAGCAAGTTAAAATAAGGCTAGTC | Intermediate primers for preparing the in vitro sgRNA template |
| sgRNA-R | AAAAGCACCGACTCGGTGCCACTTTTTCAAGTTGATAACGGACTAGCCTTATTTTAACT | End primers for preparing the in vitro sgRNA template |
| gd6-sgRNA-P1 | TAATACGACTCACTATAGTTGCGGATCATAGCATGGCGTTTTAGAGCTAGAAATAGCAA | Prime for sgRNA cleaved to gd6 position 1 |
| gd6-sgRNA-P2 | TAATACGACTCACTATAGTGGATTCAACGATTGAGCGGTTTTAGAGCTAGAAATAGCAA | Prime for sgRNA cleaved to gd6 position 2 |
| gd7-sgRNA-P1 | TAATACGACTCACTATAGTCATCCTCGCTTGTAGCAGGTTTTAGAGCTAGAAATAGCAA | Prime for sgRNA cleaved to gd7 position 1 |
| gd7-sgRNA-P2 | TAATACGACTCACTATAGCCAATGCCGACAATGGCTAGTTTTAGAGCTAGAAATAGCAA | Prime for sgRNA cleaved to gd7 position 2 |
| gd5-sgRNA-P1 | TAATACGACTCACTATAGCAAATCTTCCCCGCAGTCAGTTTTAGAGCTAGAAATAGCAA | Prime for sgRNA cleaved to gd5 position 1 |
| gd5-sgRNA-P2 | TAATACGACTCACTATAGACCGAAATGATGGCTGCCGGTTTTAGAGCTAGAAATAGCAA | Prime for sgRNA cleaved to gd5 position 2 |
| gd4-sgRNA-P1 | TAATACGACTCACTATAGTGGCTCGTTATCATATGTCGTTTTAGAGCTAGAAATAGCAA | Prime for sgRNA cleaved to gd4 position 1 |
| gd4-sgRNA-P2 | TAATACGACTCACTATAGATGCAATCAACTGGGGCCGGTTTTAGAGCTAGAAATAGCAA | Prime for sgRNA cleaved to gd4 position 2 |
| gd10-sgRNA-P1 | TAATACGACTCACTATAGCCACTACCAACGACCGCGAGTTTTAGAGCTAGAAATAGCAA | Prime for sgRNA cleaved to gd10 position 1 |
| gd10-sgRNA-P2 | TAATACGACTCACTATAGGATTGAGGCACGAAGCTTGGTTTTAGAGCTAGAAATAGCAA | Prime for sgRNA cleaved to gd10 position 2 |
| gd8-sgRNA-P1 | TAATACGACTCACTATAGATTGATCGAAGCGCTCTGTGTTTTAGAGCTAGAAATAGCAA | Prime for sgRNA cleaved to gd8 position 1 |
| gd8-sgRNA-P2 | TAATACGACTCACTATAGACGATCAACAGCTCAAACCGTTTTAGAGCTAGAAATAGCAA | Prime for sgRNA cleaved to gd8 position 2 |

| | | |
|---|---|---|
| gd9-sgRNA-P1 | TAATACGACTCACTATAGCGTGTTTGACATTATGGGCGTTTTAGAGCTAGAAATAGCAA | Prime for sgRNA cleaved to gd9 position 1 |
| gd9-sgRNA-P2 | TAATACGACTCACTATAGTTTATGCAAGCCTGCTGCAGTTTTAGAGCTAGAAATAGCAA | Prime for sgRNA cleaved to gd9 position 2 |
| gd1-sgRNA-P1 | TAATACGACTCACTATAGGTGTAGACCAACCGGAGCCGTTTTAGAGCTAGAAATAGCAA | Prime for sgRNA cleaved to gd1 position 1 |
| gd1-sgRNA-P2 | TAATACGACTCACTATAGCTTCTTACGTAGATGCTTAGTTTTAGAGCTAGAAATAGCAA | Prime for sgRNA cleaved to gd1 position 2 |
| gd3-sgRNA-P1 | TAATACGACTCACTATAGCCAAGCCAGTGATGGCAATGTTTTAGAGCTAGAAATAGCAA | Prime for sgRNA cleaved to gd3 position 1 |
| gd3-sgRNA-P2 | TAATACGACTCACTATAGTTTGTCTCATATAACGCATGTTTTAGAGCTAGAAATAGCAA | Prime for sgRNA cleaved to gd3 position 2 |
| gd2-sgRNA-P1 | TAATACGACTCACTATAGGTATGGCTTGCAAGTTCCCGTTTTAGAGCTAGAAATAGCAA | Prime for sgRNA cleaved to gd2 position 1 |
| gd2-sgRNA-P2 | TAATACGACTCACTATAGAAAGAGTCCAATGTGGTTCGTTTTAGAGCTAGAAATAGCAA | Prime for sgRNA cleaved to gd2 position 2 |
| ins3-sgRNA-P | TAATACGACTCACTATAGCGGCAGTCAACTAAGGCAAGTTTTAGAGCTAGAAATAGCAA | Prime for sgRNA cleaved to ins3 position |
| ins4-sgRNA-P | TAATACGACTCACTATAGAAATGCAGACGACCGACAAGTTTTAGAGCTAGAAATAGCAA | Prime for sgRNA cleaved to ins4 position |
| ins2-sgRNA-P | TAATACGACTCACTATAGTTCACCTCCGAGACTTACCGTTTTAGAGCTAGAAATAGCAA | Prime for sgRNA cleaved to ins2 position |
| ins1-sgRNA-P | TAATACGACTCACTATAGGACAAGATTGGCAAGTGCGGTTTTAGAGCTAGAAATAGCAA | Prime for sgRNA cleaved to ins1 position |
| OE Ins5-sgRNA-P | TAATACGACTCACTATAGATTGCCTTGGCGAAGAAGTGTTTTAGAGCTAGAAATAGCAA | Prime for sgRNA cleaved to Gd11 position 1 |
| cut Gd11 sgRNA-P | TAATACGACTCACTATAGACCAACAAGATCTGCCACCGTTTTAGAGCTAGAAATAGCAA | Prime for sgRNA cleaved to Gd11 position 1 |
| ins6-sgRNA-P | TAATACGACTCACTATAGGCAAAGTAGTGGCCTGCAAGTTTTAGAGCTAGAAATAGCAA | Prime for sgRNA cleaved to ins6 |

| | | |
|---|---|---|
| | | position |
| gd6-5F-F | GAGCCACTTTTGATTCACGGAAC | Upstream primer for 5F of gd6 |
| gd6-5F-R | GTTCCTATTCCGAAGTTCCTATTCTCTAGAGTTGATACTAGGTTCAGGTCATGAATTTG | Downstream primer for 5F of gd6 |
| gd6-3F-F | GCCTGAATGGCGAATGGAAATTGTAAGCGTTAATCTAGACGGTGCGTTGTATCTACCTG | Upstream primer for 3F of gd6 |
| gd6-3F-R | GGCACATACGGAAGGACTTG | Downstream primer for 3F of gd6 |
| gd6-nest-F | GACGCACCTTATACGGAAGATC | For whole length of KO cassette |
| gd6-nest-R | AATCATGATCACGGACTCGATAC | For whole length of KO cassette |
| gd6-RT-F | CGAGTTGAGGACGACATCG | Validation primer for gd6 |
| gd6-RT-R | CAACACAATGCGCTGATCCC | Validation primer for gd6 |
| gd7-5F-F | CCTCCACAGGAAGATGATGC | Upstream primer for 5F of gd7 |
| gd7-5F-R | GCTTTGAAGTTCCTATTCCGAAGTTCCTATTCTCTAGACGTCACGTGGTATAATTGGCC | Downstream primer for 5F of gd7 |
| gd7-3F-F | GAATGGAAATTGTAAGCGTTAATCTAGAGTTTTATTGTAATGGAATGCACAAAGAAATT | Upstream primer for 3F of gd7 |
| gd7-3F-R | AACAGACATTTGTATTCAGCTGTCATG | Downstream primer for 3F of gd7 |
| gd7-nest-F | GGCGAGGAGTGACAGGAAC | For whole length of KO cassette |
| gd7-nest-R | GACGCGTTTATCTAGAAGGATGT | For whole length of KO cassette |
| gd7-RT-F | CTGTTGCTATCGCGACTTGTC | Validation primer for gd7 |
| gd7-RT-R | CGCTTCATTGCTGCTTGC | Validation primer for gd7 |
| gd5-5F-F | TGCAATCTGTGTCACCGAC | Upstream primer for 5F of gd5 |
| gd5-5F-R | TTGAAGTTCCTATTCCGAAGTTCCTATTCTCTAGACTACAGCGAGGGTTTCTTATGTAC | Downstream primer for 5F of gd5 |
| gd5-3F-F | GAATGGCGAATGGAAATTGTAAGCGTTAATCTAGATTCGGTCAACACGGGACTTAATTG | Upstream primer for 3F of gd5 |
| gd5-3F-R | ATAGGAGCTGAGCCGCAC | Downstream primer for 3F of gd5 |
| gd5-nest-F | ACGCGACTATAGCTACATTTCAAG | For whole length of KO cassette |
| gd5-nest-R | TCGTCAAAGCTAAGAGTCGCAG | For whole length of |

| | | |
|---|---|---|
| | | KO cassette |
| gd5-RT-F | CAAAAGCGCTCTTGAACCATC | Validation primer for gd5 |
| gd5-RT-R | AATGGTATCTTGACGAGGTCCTG | Validation primer for gd5 |
| gd4-5F-F | AGATCGTAGGTATCAGATGGGAG | Upstream primer for 5F of gd4 |
| gd4-5F-R | TGAAGTTCCTATTCCGAAGTTCCTATTCTCTAGAG ATGAAGATATCTTGGAGCAGGAGG | Downstream primer for 5F of gd4 |
| gd4-3F-F | CTGAATGGCGAATGGAAATTGTAAGCGTTAATCT AGAGGGCTATTTCATTCCAAGCTTG | Upstream primer for 3F of gd4 |
| gd4-3F-R | TGCAAATGAGCCGTTGCC | Downstream primer for 3F of gd4 |
| gd4-nest-F | TTCTCGAAAAGTGACGTAGTTCATC | For whole length of KO cassette |
| gd4-nest-R | TGCTGAATAGACATGGTGGATATGG | For whole length of KO cassette |
| gd4-RT-F | CAAGAATCTTATCCCCATCTTTTCCAG | Validation primer for gd4 |
| gd4-RT-R | AGTGCCAGTTCCATGCATC | Validation primer for gd4 |
| gd10-5F-F | TACCAATTCGGCACGTTTCAC | Upstream primer for 5F of gd10 |
| gd10-5F-R | AGTTCCTATTCCGAAGTTCCTATTCTCTAGAAGTA TCTTGAGTAAATGTGTTAAGTTGG | Downstream primer for 5F of gd10 |
| gd10-3F-F | CAGCCTGAATGGCGAATGGAAATTGTAAGCGTTA ATCTAGAGGCACGAAGCTTGAGGAG | Upstream primer for 3F of gd10 |
| gd10-3F-R | GGCGAAGCTGTCTATTTTCGAG | Downstream primer for 3F of gd10 |
| gd10-nest-F | TTGCCCCGTATATGTAAGCCTC | For whole length of KO cassette |
| gd10-nest-R | TTTGACCCCAAATCACTTCTATTGAC | For whole length of KO cassette |
| gd10-RT-F | CGATTGCAACGAGCTCTGAC | Validation primer for gd10 |
| gd10-RT-R | CATACCCATAACGACGTAGCC | Validation primer for gd10 |
| gd8-5F-F | ATATCGGAGATGCCAAGATTGTTG | Upstream primer for 5F of gd8 |
| gd8-5F-R | AACGCTTTGAAGTTCCTATTCCGAAGTTCCTATTC TCTAGAGTAACAAATGGGGGCGCT | Downstream primer for 5F of gd8 |
| gd8-3F-F | CGAATGGAAATTGTAAGCGTTAATCTAGAGATGA TGTAGTCTAAGAATCTATGTGACAC | Upstream primer for 3F of gd8 |
| gd8-3F-R | GGTAAATTCGGTGGCTCCTTG | Downstream primer |

| | | |
|---|---|---|
| | | for 3F of gd8 |
| gd8-nest-F | ATCTTCTCGAGGAATCCGGC | For whole length of KO cassette |
| gd8-nest-R | TCGCAGGCATTGTGATGAGTAG | For whole length of KO cassette |
| gd8-RT-F | CAAAGGCGTCCTCGAACG | Validation primer for gd8 |
| gd8-RT-R | TAAAATCCCTGGCGGCTTATCC | Validation primer for gd8 |
| gd9-5F-F | CCCAGCAGGTTCTTTCGAG | Upstream primer for 5F of gd9 |
| gd9-5F-R | GCTTTGAAGTTCCTATTCCGAAGTTCCTATTCTCT AGAGAAGCGTATGGCAATTCGTTG | Downstream primer for 5F of gd9 |
| gd9-3F-F | CAGCCTGAATGGCGAATGGAAATTGTAAGCGTTA ATCTAGAGATTGCCTTGCAGCAGGC | Upstream primer for 3F of gd9 |
| gd9-3F-R | AGGACCAGGTCTTGGTGG | Downstream primer for 3F of gd9 |
| gd9-nest-F | AAGGAGGTTTTGTCGAGACTCAG | For whole length of KO cassette |
| gd9-nest-R | GACAGAGATCCCGGAAGATTTC | For whole length of KO cassette |
| gd9-RT-F | TTTGATTGGTGGCATGTTTCGC | Validation primer for gd9 |
| gd9-RT-R | AGTCAAGTTGGATATTCCGGTCC | Validation primer for gd9 |
| gd1-5F-F | CGGACCCAATGTATAATTTGAAGC | Upstream primer for 5F of gd1 |
| gd1-5F-R | AAGTTCCTATTCCGAAGTTCCTATTCTCTAGATGT AGCAACTGTTGCTCGAAATATCAG | Downstream primer for 5F of gd1 |
| gd1-3F-F | ATGGAAATTGTAAGCGTTAATCTAGATTTCTGTAT AGTCATATATTCTTGCTTCTTACG | Upstream primer for 3F of gd1 |
| gd1-3F-R | GAAGAATGTCACGTCACTATGCAG | Downstream primer for 3F of gd1 |
| gd1-nest-F | ATCATGGTTGCAGTCAGCAG | For whole length of KO cassette |
| gd1-nest-R | AAGATAATCGGTGAGCAATGATGG | For whole length of KO cassette |
| gd1-RT-F | GCAGGACTCTTCCATCTCTTCC | Validation primer for gd1 |
| gd1-RT-R | TTTGTCAGAGATCCACTCGGC | Validation primer for gd1 |
| gd3-5F-F | GTAGTAGATGCATACGTCAAATTCAATAC | Upstream primer for 5F of gd3 |
| gd3-5F-R | GAAGTTCCTATTCCGAAGTTCCTATTCTCTAGATT | Downstream primer |

| | | |
|---|---|---|
| | TGAGCTACTTGAGAAAATGGGTTG | for 5F of gd3 |
| gd3-3F-F | TGGCGAATGGAAATTGTAAGCGTTAATCTAGATTTGGCTCTACTGATGATGCTATTGAG | Upstream primer for 3F of gd3 |
| gd3-3F-R | GCTGGGTGAACCTTTATGCT | Downstream primer for 3F of gd3 |
| gd3-nest-F | CATAGATATCAGGCCCGATGC | For whole length of KO cassette |
| gd3-nest-R | AAAGGATTGGGGTAATGGCAG | For whole length of KO cassette |
| gd3-RT-F | TCCCGAGAAGTTATGGGAGGTATG | Validation primer for gd3 |
| gd3-RT-R | TTACTGATTGAGGCTCTACGCAC | Validation primer for gd3 |
| gd2-5F-F | ATCTACGCCAAGCCTTGG | Upstream primer for 5F of gd2 |
| gd2-5F-R | TGAAGTTCCTATTCCGAAGTTCCTATTCTCTAGAATTGAATATGCGGCAAAGATGATAC | Downstream primer for 5F of gd2 |
| gd2-3F-F | ATGGCGAATGGAAATTGTAAGCGTTAATCTAGAAATGACGAATAATGATTGAGGGATTG | Upstream primer for 3F of gd2 |
| gd2-3F-R | TGGGCTCTGCCGGAAATATG | Downstream primer for 3F of gd2 |
| gd2-nest-F | CCAGCTGGCACTTTTGGATAATC | For whole length of KO cassette |
| gd2-nest-R | ATCCGTAGATCACGAAAGAGACG | For whole length of KO cassette |
| gd2-RT-F | AACATTGGCCATTCCGAGG | Validation primer for gd2 |
| gd2-RT-R | GCTCATGATGGAGGTGTCG | Validation primer for gd2 |
| ins2-5F-F | CATGCCAGGTGATTGGAGG | Upstream primer for 5F of ins2 |
| ins2-5F-R | CCTGAATGGCGAATGGAAATTGTAAGCGTTAATCTAGAAAGTCTCGGAGGTGAACGAAG | Downstream primer for 5F of ins2 |
| ins2-3F-F | AGCTTGACTAACAGCTACCCCGCTTGAGCAGACATCACCATGGAATTGACCGACGATGC | Upstream primer for 3F of ins2 |
| ins2-3F-R | CAACATCTGTAATCGATAGCTCG | Downstream primer for 3F of ins2 |
| ins2-Nest-F | GTACTCGCAGTCCAGTGTCATC | For whole length of insertion cassette |
| ins2-Nest-R | CGAAGTGATGAAACGGTGGAC | For whole length of insertion cassette |
| ins1-5F-F | CACGGATAATCCATATTGAGCGC | Upstream primer for 5F of ins1 |
| ins1-5F-R | GCCTGAATGGCGAATGGAAATTGTAAGCGTTAAT | Downstream primer |

| | | |
|---|---|---|
| | CTAGAGCGAGGAATATAGGCGATGG | for 5F of ins1 |
| ins1-3F-F | CAGCTACCCCGCTTGAGCAGACATCACCATGTCATTCTCAAATTTCTTTCAATATTCGG | Upstream primer for 3F of ins1 |
| ins1-3F-R | GTGGCTCGGGAGTCTATG | Downstream primer for 3F of ins1 |
| ins1-nest-F | CCTAGTATTCCGCTAGTAGCATGG | For whole length of insertion cassette |
| ins1-nest-R | CCGGCAAGAGTTTCAACTCC | For whole length of insertion cassette |
| ins3-5F-F | CCGCCATTTGTTCTATGTTCTACC | Upstream primer for 5F of ins3 |
| ins3-5F-R | GCCTGAATGGCGAATGGAAATTGTAAGCGTTAATCTAGACCTTAGTTGACTGCCGCAAG | Downstream primer for 5F of ins3 |
| ins3-3F-F | CGCAGCTTGACTAACAGCTACCCCGCTTGAGCAGACATCACCATGGCCACCACCAACAC | Upstream primer for 3F of ins3 |
| ins3-3F-R | CCATCCGAAAGTTCTCCACC | Downstream primer for 3F of ins3 |
| ins3-nest-F | CACCTCCGACTTGCCTTTATC | For whole length of insertion cassette |
| ins3-nest-R | GACCAAATGTTGGGCGTTG | For whole length of insertion cassette |
| ins4-5F-F | CGCACCTCTCCGGAATAAC | Upstream primer for 5F of ins4 |
| ins4-5F-R | CCTGAATGGCGAATGGAAATTGTAAGCGTTAATCTAGACAACGGATGGCTTGTACATTC | Downstream primer for 5F of ins4 |
| ins4-3F-F | TGACTAACAGCTACCCCGCTTGAGCAGACATCACCATGTTGCAAACATCTCAAAGGTAC | Upstream primer for 3F of ins4 |
| ins4-3F-R | GTTCGGTAGCCCGTCGTAC | Downstream primer for 3F of ins4 |
| ins4-nest-F | GAGAGCGTCATGCAATTCTTGG | For whole length of insertion cassette |
| ins4-nest-R | CGCGTCTTCTCGGCTATAATAC | For whole length of insertion cassette |
| G418::gpdA-F | ACGCTTTGAAGTTCCTATTCCGAAGTTCCTATTCTCTAGACCGATAGCTCTGCAAAGGG | A primer linking *neo* and gpdA promoter |
| Cut Gd11-5F-F | GTCGGTACGAAAGGCTATTCATC | Upstream primer for 5F of Gd11 |
| cut Gd11-5F-R | TGACTTGCATTGTGCAACGCCCTTTGCAGAGCTATCGGGGCAGATCTTGTTGGTCTTGC | Downstream primer for 5F of Gd11 |
| gpdA-F | CCGATAGCTCTGCAAAGGG | A primer for gpdA promoter |
| OE Ins5 -3F-F | CAGCTTGACTAACAGCTACCCCGCTTGAGCAGACATCACCATGTCGCCACCCCATAGTG | Upstream primer for 3F of Ins5 |
| OE Ins5 -3F-R | CGCCAAGGCCATATAGCG | Downstream primer |

| | | |
|---|---|---|
| | | for 3F of Ins5 |
| cut Gd11-nest-F | GTTCTGCTGCTCATCATGGTC | For whole length of KO cassette |
| OE Ins5 -nest-R | CGCAAGCATAAAATTAAGACGC | For whole length of KO cassette |
| OE ins6-5F-F | CCTCAGCTACTGCATATGTTACC | Upstream primer for 5F of ins6 |
| OE ins6-5F-R | CAGCCTGAATGGCGAATGGAAATTGTAAGCGTTAATCTAGACAGGCCACTACTTTGCCC | Downstream primer for 5F of ins6 |
| Gd11 Pro-F | AACGCTTTGAAGTTCCTATTCCGAAGTTCCTATTCTCTAGATGGGATCGTGGCTAAAAG | Primer for 7101 promoter |
| Gd11 Pro-R | TATGTAAGGCGCGGCTCAG | Primer for 7101 promoter |
| ins6-3F-F | CGACTTCACTCAGATACTGAGCCGCGCCTTACATAATGTCTGTATCTAGTAAAGACGGC | Upstream primer for 3F of ins6 |
| ins6-3F-R | CGGATGCGAAGTATAGAGGTTC | Downstream primer for 3F of ins6 |
| ins6-nest-F | GAGGATATCGTCCTCGCCG | For whole length of insertion cassette |
| ins6-nest-R | CTCAAGAGGGTCACCGACAG | For whole length of insertion cassette |
| G418-detdct-5F-R | GTAGCCAACGCTATGTCCTG | Validation primer |
| G418-detect-3F-F | CATCAGACAGTACATGCATGTTGC | Validation primer |
